# miR-135b Promotes Cancer Progression by Targeting Transforming Growth Factor Beta Receptor II (TGFBR2) in Colorectal Cancer

**DOI:** 10.1371/journal.pone.0130194

**Published:** 2015-06-10

**Authors:** Jialu Li, Hongwei Liang, Ming Bai, Tao Ning, Cheng Wang, Qian Fan, Yanbo Wang, Zheng Fu, Nan Wang, Rui Liu, Ke Zen, Chen-Yu Zhang, Xi Chen, Yi Ba

**Affiliations:** 1 Tianjin Medical University Cancer Institute and Hospital, Key Laboratory of Cancer Prevention and Therapy, Tiyuanbei, Tianjin, 300060, China; 2 Jiangsu Engineering Research Center for microRNA Biology and Biotechnology, State Key Laboratory of Pharmaceutical Biotechnology, School of Life Sciences, Nanjing University, Nanjing, 210093, China; 3 Department of Clinical Laboratory, Jinling Hospital, Clinical School of Medical College, Nanjing University, Nanjing, China; University of Kansas School of Medicine, UNITED STATES

## Abstract

The transforming growth factor beta (TGF-β) signaling pathway is a tumor-suppressor pathway that is commonly inactivated in colorectal cancer (CRC). The inactivation of TGFBR2 is the most common genetic event affecting the TGF-β signaling pathway. However, the mechanism by which cancer cells downregulate TGFBR2 is unclear. In this study, we found that the TGFBR2 protein levels were consistently upregulated in CRC tissues, whereas its mRNA levels varied in these tissues, suggesting that a post-transcriptional mechanism is involved in the regulation of TGFBR2. Because microRNAs (miRNAs) are powerful post-transcriptional regulators of gene expression, we performed bioinformatic analyses to search for miRNAs that potentially target TGFBR2. We identified the specific targeting site of miR-135b in the 3’-untranslated region (3’-UTR) of TGFBR2. We further identified an inverse correlation between the levels of miR-135b and TGFBR2 protein, but not mRNA, in CRC tissue samples. By overexpressing or silencing miR-135b in CRC cells, we experimentally validated that miR-135b directly binds to the 3’-UTR of the TGFBR2 transcript and regulates TGFBR2 expression. Furthermore, the biological consequences of the targeting of TGFBR2 by miR-135b were examined using in vitro cell proliferation and apoptosis assays. We demonstrated that miR-135b exerted a tumor-promoting effect by inducing the proliferation and inhibiting the apoptosis of CRC cells via the negative regulation of TGFBR2 expression. Taken together, our findings provide the first evidence supporting the role of miR-135b as an oncogene in CRC via the inhibition of TGFBR2 translation.

## Introduction

Colorectal cancer (CRC) is currently the third most common malignancy and the second leading cause of cancer-related death worldwide [1]. The accumulation of genetic and epigenetic alterations mediates CRC formation and progression by deregulating key signaling pathways in cancer cells [2,3]. In CRC, one of the most commonly inactivated signaling pathways is the transforming growth factor beta (TGF-β) signaling pathway, which has been associated with the establishment and progression of intestinal neoplasms [4].

The TGF-β signaling pathway plays important roles in many cellular processes, including cell cycle regulation, cell migration, apoptosis, and immune modulation via two related transmembrane serine/threonine kinase receptors, the type I and type II serine/threonine kinase receptors [5]. TGF-β signaling is initiated when the ligand binds to the type II receptor, which is followed by the dimerization of the type II receptor with the type I receptor. Within this heteromeric complex, the type II receptor phosphorylates and activates the type I receptor kinase, which propagates the signal by targeting downstream components of this pathway [6]. The TGF-β signaling pathway acts as a tumor-suppressor during the early stage of CRC, which is often inactivated via the downregulation of TGFBR2 [7]. A decrease in the TGFBR2 expression levels has been associated with increased tumorigenicity in a number of human tumors, including CRC [8]. The inactivation of TGBR2 due to genetic alteration or promoter methylation has been reported in esophageal, gastric and prostate cancers [9–11]. The inactivation of TGFBR2 due to genetic mutation or methylation was reported to primarily occur in microsatellite-instable CRC because of DNA mismatch repair defects [12–14]. However, tumors exhibiting microsatellite instability only account for 10–15% of all CRC cases [15]. The mechanism underlying non-mismatch repair-deficient CRC remains unclear. These observations suggest that other molecular mechanisms may be involved in the downregulation of TGFBR2; this hypothesis requires further investigation.

MicroRNAs (miRNAs) are a class of small non-coding, single-stranded RNAs that play an important role in the regulation of gene expression at the post-transcriptional level [16–18]. Recent evidence has indicated that miRNAs can function as oncogenes or tumor suppressors by repressing cancer-related genes [19]. Alterations of miRNA expression have been observed in a variety of human tumors, including CRC [20,21]. Some of these miRNAs have attracted special attention due to their involvement in the initiation, progression, and metastasis of human cancers [22,23]. For example, miR-143 and miR-145 (miR-143/145) are downregulated in many types of cancer, including CRC [24,25]. Moreover, it was reported that miR-143/145 act as tumor suppressors via the inhibition of KRAS translation in human CRC [26–28]. These findings underscore the need for an in-depth search for miRNAs that are aberrantly expressed during colorectal carcinogenesis and the need for an intensive investigation of their role in tumor biology.

Although the deregulation of TGFBR2 and miRNAs is associated with tumorigenesis in human CRC, little is known about which miRNAs act on TGFBR2. In this study, we hypothesized that TGFBR2 is a target of miR-135b. After measuring the expression levels of miR-135b and TGFBR2 in CRC tissues and paired noncancerous tissues, we detected an inverse correlation between miR-135b and TGFBR2 expression in CRC. Furthermore, in this study, we experimentally confirmed the direct regulation of TGFBR2 by miR-135b and the biological role of the miR-135b-mediated regulation of TGFBR2 expression in human CRC.

## Materials and Methods

### Human tissue

CRC tissues and paired adjacent noncancerous tissues were obtained from patients undergoing surgical procedures at the Affiliated Gulou Hospital of Nanjing University (Nanjing, China). Both the tumor and noncancerous tissues were subjected to histological analysis for diagnostic confirmation. The pathological type of each cancer was identified as adenocarcinoma. Written consent was provided by all of the patients or their guardians, and the Ethics Committee of The First Affiliated Hospital of Anhui Medical University approved all aspects of this study. Tissue fragments were immediately frozen in liquid nitrogen at the time of surgery and were stored at -80°C. The clinical characteristics of the patients are listed in S1 Table.

### Cell culture

The human CRC cell lines HT-29 and SW-480 were purchased from the Shanghai Institute of Cell Biology of the Chinese Academy of Sciences (Shanghai, China). The HT-29 and SW-480 cells were cultured in RPMI-1640 medium (Gibco, Carlsbad, CA, USA) supplemented with 10% fetal bovine serum (Gibco) in a humidified incubator at 37°C in 5% CO_2_.

### RNA isolation and quantitative RT-PCR

Total RNA was extracted from the cultured cells and tissues using Trizol reagent (Invitrogen) according to the manufacturer’s instructions. Assays to quantify mature miRNAs were performed using TaqMan miRNA probes (Applied Biosystems, Foster City, CA, USA) according to the manufacturer’s instructions. Briefly, 1 μg of total RNA was reverse-transcribed to cDNA using AMV reverse transcriptase (TaKaRa, Dalian, China) and a stem-loop RT primer (Applied Biosystems). The reaction conditions were as follows: 16°C for 30 min, 42°C for 30 min and 85°C for 5 min. Real-time PCR was performed using a TaqMan PCR kit and an Applied Biosystems 7500 Sequence Detection System (Applied Biosystems). The reactions were incubated in a 96-well optical plate at 95°C for 5 min, followed by 40 cycles of 95°C for 15 s and 60°C for 1 min. All of the reactions were performed in triplicate. After the reactions were complete, the cycle threshold (C_T_) data were collected using fixed threshold settings, and the mean C_T_ was determined from triplicate PCRs. A comparative C_T_ method was used to compare each transcript to the controls. U6 snRNA was used as an internal control, and the relative amount of miRNA normalized to the U6 snRNA levels was calculated using the formula 2^-ΔΔCT^, in which ΔΔC_T_ = (C_T miRNA_ − C_T U6_)_target_ − (C_T miRNA_ − C_T U6_)_control_.

To quantify the TGFBR2 and GAPDH mRNA levels, 1 μg of total RNA was reverse-transcribed to cDNA using oligo d(T)18 primers (TaKaRa) and ThermoScript reverse transcriptase (Invitrogen). The reaction conditions were as follows: 42°C for 60 min and 70°C for 10 min. Real-time PCR was then performed on the RT product, SYBR Green dye (Invitrogen) and specific primers for TGFBR2 or GAPDH. The sequences of the primers were as follows: TGFBR2 sense: 5’-GTAGCTCTGATGAGTGCAATGAC-3’; TGFBR2 antisense: 5’-CAGATATGGCAACTCCCAGTG-3’; GAPDH sense: 5’-GATATTGTTGCCATCAATGAC-3’; and GAPDH antisense: 5’-TTGATTTTGGAGGGATCTCG-3’. The reactions were incubated at 95°C for 5 min, followed by 40 cycles of 95°C for 30 s, 55°C for 30 s and 72°C for 1 min. After the reactions were completed, the C_T_ values were determined according to a fixed threshold. The relative amount of TGFBR2 mRNA was normalized to the GAPDH mRNA level.

### miRNA overexpression

miRNA overexpression was achieved by transfecting cells with a miRNA mimic, which is a synthetic RNA oligonucleotide duplex mimicking the miRNA precursor sequence. Synthetic RNA molecules, including pre-miR-135b and a scrambled negative control RNA (pre-miR-control), were purchased from GenePharma (Shanghai, China). HT-29 and SW-480 cells were seeded on 6-well plates and were transfected using Lipofectamine 2000 (Invitrogen) on the following day, when the cells were approximately 70% confluent. For the miRNA overexpression experiments, 100 pmol of pre-miR-135b were used. After 6 h, the medium of the HT-29 and SW-480 cells was changed to RPMI-1640 medium supplemented with 2% fetal bovine serum. The cells were harvested at 24 h or 48 h post-transfection for the isolation of total RNA or protein, respectively.

### Plasmid construction and siRNA efficiency assay

A mammalian expression plasmid (pReceiver-M98-TGFBR2) designed to specifically express the full-length open reading frame (ORF) of human TGFBR2 without the miR-135b—responsive 3’-UTR was purchased from FulenGen (Guangzhou, China). An empty plasmid served as a negative control. The siRNA sequence (5′-GATTCAAGAGTATTCTCACTT-3’) targeting human TGFBR2 was designed and synthesized by Ribobio (Guangzhou, China). A scrambled siRNA (Ribobio) was used as a negative control. The overexpression plasmid or siRNA was transfected into HT-29 using Lipofectamine 2000 (Invitrogen) according to the manufacturer’s instructions. Total RNA or protein was isolated 24 h or 48 h after transfection. The TGFBR2 mRNA and protein expression levels were assessed via quantitative RT-PCR and Western blot, respectively.

### Luciferase reporter assay

The entire 3’-UTR of human TGFBR2 was amplified via PCR using human genomic DNA as a template. The PCR products were then inserted into the pMIR-REPORT plasmid (Ambion, Austin, TX, USA). The successful insertion of this sequence was confirmed via DNA sequencing. To evaluate binding specificity, the sequences that interact with the seed region of miR-135b were mutated (from AGCUAUG to UCGAUAC for the miR-135b binding site), and the mutant TGFBR2 3’-UTR was inserted into an equivalent luciferase reporter plasmid. For luciferase reporter assays, HT-29 and SW-480 cells were seeded on 24-well plates and co-transfected with 0.8 μg of the firefly luciferase reporter plasmid, 0.8 μg of the β-galactosidase (β-gal) expression plasmid (Ambion), and equal amounts (20 pmol) of the miR-135b mimic or a scrambled negative control RNA using Lipofectamine 2000 (Invitrogen). The β-gal plasmid was used as a transfection efficiency control. The cells were harvested 24 h after transfection and were subjected to the luciferase activity assay using a luciferase assay kit (Promega, Madison, WI, USA).

### Protein isolation and Western blot

Cells or tissues were lysed in RIPA lysis buffer (50 mM Tris-HCl, 150 mM NaCl, 0.1% SDS, 1% NP-40, 0.25% sodium deoxycholate and 1 mM EDTA, pH 8.0) containing freshly added protease inhibitor cocktail (Roche) for 30 min on ice and were then centrifuged at 16,000 × g at 4°C for 10 min. The supernatant was collected, and the protein concentration was calculated using a BCA protein assay kit (Thermo Scientific, Rockford, IL, USA). The proteins were separated via SDS-PAGE (Bio-Rad). After electrophoresis, the proteins were electrotransferred to PVDF membranes (Bio-Rad) and then blocked with 5% skim milk for 1 h. The membranes were then incubated in primary antibodies at 4°C for 12 h. After three washes in TBST, the membranes were incubated in a horseradish peroxidase-conjugated secondary antibody for 1 h at room temperature. After three washes, the membranes were incubated in the SuperSignal West Pico chemiluminescence substrate (Pierce). The same membrane was probed with a GAPDH antibody to serve as a loading control. The antibodies against TGFBR2 and GAPDH were purchased from Santa Cruz Biotechnology (sc-400 and sc-365062, respectively; Santa Cruz, CA, USA).

### Cell proliferation assay

HT-29 cells were seeded at 5 × 10^4^ cells per well on 96-well plates and then incubated overnight in RPMI-1640 supplemented with 10% FBS. The cells were collected at 12, 24, 36, 48 or 60 h post-transfection. After transfection, 10 μL of Cell Counting Kit-8 solution (CK04-500, Dojindo) was added to the corresponding tested wells and incubated for 1 h. The absorbance was measured at a wavelength of 450 nm. All experiments were performed in triplicate.

### Apoptosis assays

The apoptosis of HT-29 cells was assessed using an Annexin V-FITC/propidium iodide (PI) staining assay. The HT-29 cells were cultured in 12-well plates and transfected with pre-miR-135b, anti-miR-135b, TGFBR2 siRNA, or the TGFBR2 overexpression plasmid to induce apoptosis. Pre-miR-control, anti-miR-control, control siRNA, and a control plasmid served as negative controls. The cells were cultured for 24 h in serum-depleted medium; then, the attached and floating cells were harvested. Flow cytometric analysis of the apoptotic cells was performed using an Annexin V-FITC/PI staining kit (BD Biosciences, CA, USA). After washing with cold PBS, the cells were resuspended in binding buffer (100 mM HEPES, pH 7.4, 100 mM NaCl, and 25 mM CaCl_2_), followed by staining with Annexin V-FITC/PI at room temperature in the dark for 15 min. The apoptotic cells were then evaluated by gating the PI- and Annexin V-positive cells using a fluorescence-activated cell-sorting (FACS) flow cytometer (BD Biosciences, San Jose, CA, USA). All experiments were performed in triplicate.

Furthermore, nuclei were stained with DAPI to evaluate morphological changes in apoptotic cells. Briefly, after transfection and culture for 24 h in serum-depleted medium, HT-29 cells were stained with DAPI for 15 min at 37°C under 5% (v/v) CO2. After washing with cold PBS and fixing in 4% (v/v) formalin, cells were examined using an Olympus IX81 microscope (200×) equipped with X-CITE fluorescence illumination (Series 120Q; blue fluorescence). The extent of apoptosis was quantified by calculating the ratio of condensed to total nuclei. Results were shown from the data of three independent experiments with 3 random microscopic fields from each sample. Condensed nuclei in the microscopic field were counted.

### Statistical analysis

All of the Western blot and proliferation assay images are representative of at least three independent experiments. The quantitative RT-PCR and luciferase reporter assays were performed in triplicate, and each individual experiment was repeated several times. The results are presented as the means ± SE of at least three independent experiments. The observed differences were considered to be significant at p < 0.05 based on Student’s t-test.

## Results

### Upregulation of the TGFBR2 protein, but not mRNA, in CRC tissues

**Fig 1:**
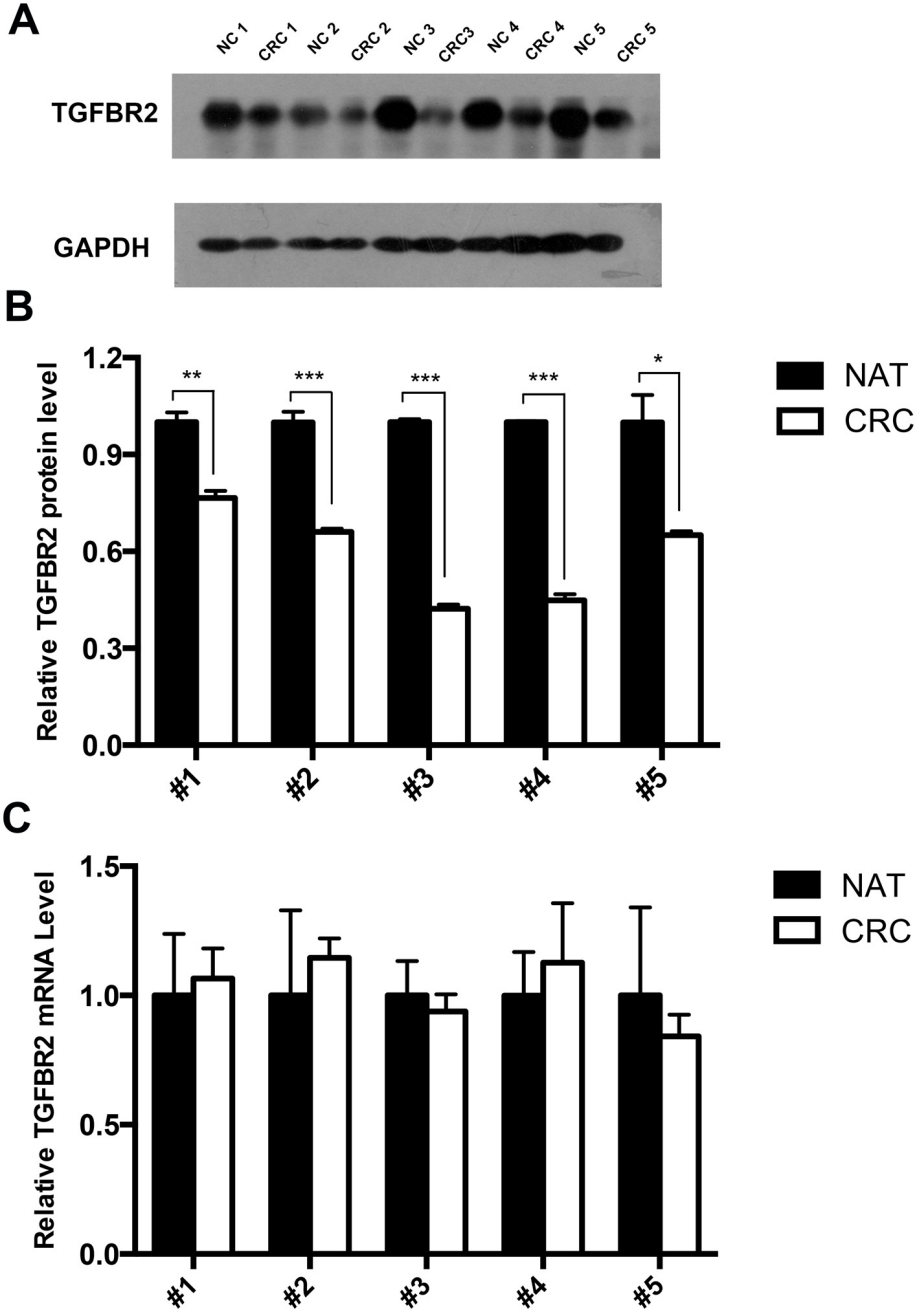
Upregulation of the TGFBR2 protein, but not mRNA, in CRC tissues. **(A and B)** Western blot analysis of the TGFBR2 protein levels in five paired CRC and normal adjacent tissue (NAT) samples. A: representative image; B: quantitative analysis. **(C)** Quantitative RT-PCR analysis of the TGFBR2 mRNA levels in five paired CRC and NAT tissue samples. * P < 0.05; ** P < 0.01; *** P < 0.001.

We first determined the expression patterns of TGFBR2 in human CRC tissues. After measuring the protein levels of TGFBR2 in five paired CRC and normal adjacent tissues, we found that the TGFBR2 protein levels were dramatically reduced in the CRC tissues compared to the normal adjacent tissues (Fig 1A and 1B). However, although the TGFBR2 protein was consistently downregulated in the CRC tissue, the TGFBR2 mRNA levels did not significantly differ between the cancerous and noncancerous tissues (Fig 1C). This disparity between the protein and mRNA expression of TGFBR2 in CRC strongly suggests that a post-transcriptional mechanism is involved in the regulation of TGFBR2.

### Identification of conserved miR-135b target sites within the 3’-UTR of TGFBR2

**Fig 2:**
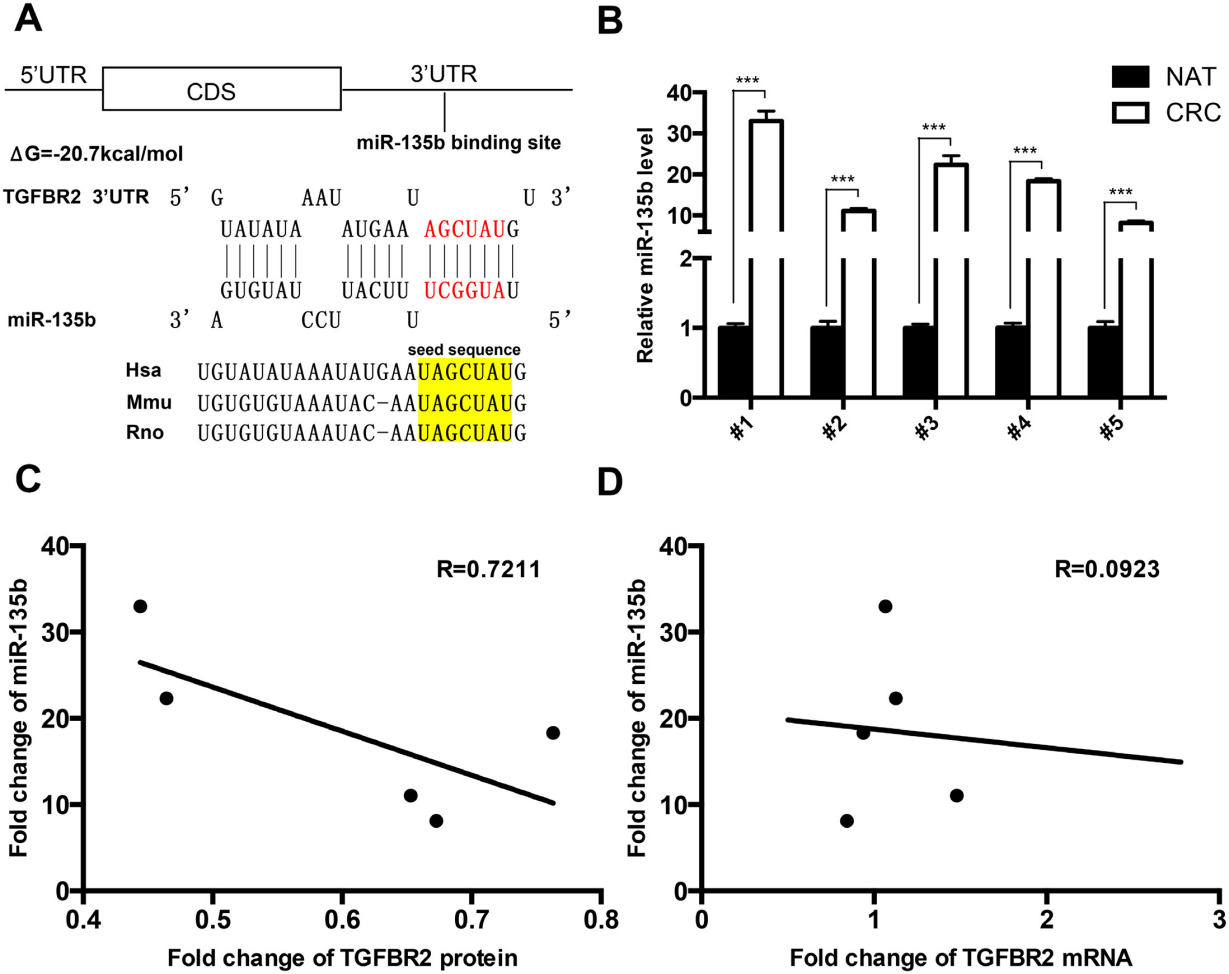
Identificatopn of an inverse correlation between the miR-135b levels and the TGFBR2 protein levels in CRC tissues. **(A)** Schematic of the hypothetical duplexes formed by the interaction between the binding sites in the TGFBR2 3’-UTR (top) and miR-135b (bottom). The predicted free energy value of this interaction is indicated. The seed recognition sites are denoted, and all nucleotides in these regions are highly conserved across several species, including human, mouse and rat. **(B)** Quantitative RT-PCR analysis of the miR-135b levels in five paired CRC and NAT tissue samples. **(C)** Pearson’s correlation scatter plot of the fold change of miR-135b and TGFBR2 protein in human CRC tissues. **(D)** Pearson’s correlation scatter plot of the fold change of miR-135b and TGFBR2 mRNA in human CRC tissues. * P < 0.05; ** P < 0.01; *** P < 0.001.

Three computational algorithms, TargetScan [29], miRanda [30] and PicTar [31], were used in combination to identify potential miRNAs that target TGFBR2. All three algorithms predicted miR-135b as a regulator of TGFBR2. The predicted interactions between miR-135b and the target sites within the 3’-UTR of TGFBR2 are illustrated in Fig 2A. One predicted hybridization was observed between miR-135b and the 3’-UTR of TGFBR2. There was perfect complementarity between the seed region (the core sequence that encompasses the first 2–8 bases of the mature miRNA) and the putative target sequence. The minimum free energy value of the hybridization between miR-135b and TGFBR2 was -20.7 kcal/mol, which is well within the range of genuine miRNA-target pairs. Furthermore, the miR-135b binding sequences in the TGFBR2 3’-UTR were highly conserved across species. Thus, miR-135b was selected for further experimental verification of its binding to TGFBR2.

### Identification of an inverse correlation between the miR-135b levels and the TGFBR2 protein levels in CRC tissues

miRNAs are generally thought to display expression patterns that are opposite to those of their targets [16–18]. Interest in the relationship between miR-135b and TGFBR2 was also supported by the recent identification of this miRNA as a candidate oncogene and of TGFBR2 as a tumor suppressor in CRC [2,32]. We next investigated whether the miR-135b levels inversely correlated with the TGFBR2 protein expression levels in CRC tissues. After determining the levels of miR-135b in five paired CRC and normal adjacent tissues, we found that the expression level of miR-135b was significantly increased in CRC tissues (Fig 2B). The inverse correlation between miR-135b and TGFBR2 protein levels (Fig 2C) and the disparity between the miR-135b and TGFBR2 mRNA levels (Fig 2D) were further illustrated using Pearson’s correlation scatter plots. These results indicated that the miR-135b expression level inversely correlates with the TGFBR2 protein expression level in human CRC tissues. Thus, TGFBR2 was determined to be a miR-135b target based on both computational predictions and the inverse correlation between the levels of miR-135b and TGFBR2 protein, but not mRNA, in CRC.

### Validation of TGFBR2 as a direct target of miR-135b

**Fig 3:**
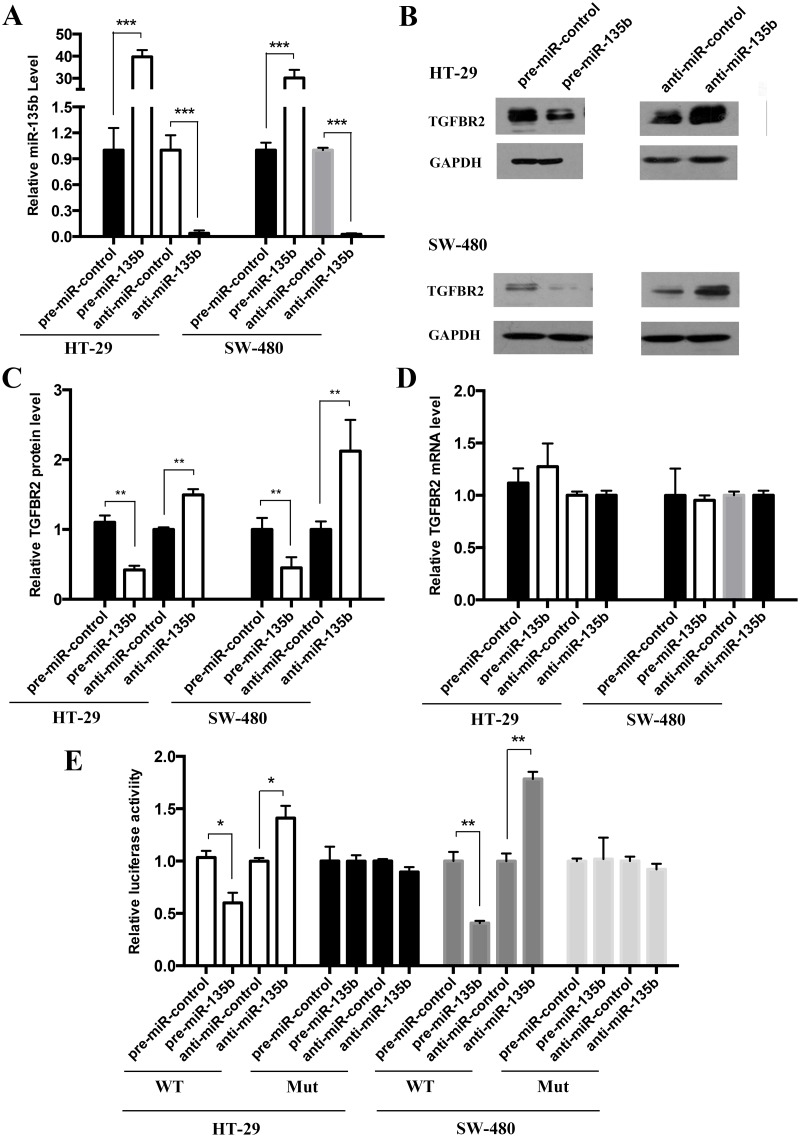
miR-135b directly regulates TGFBR2 expression at the post-transcriptional level. **(A)** Quantitative RT-PCR analysis of the miR-135b levels in HT-29 and SW-480 cells treated with scrambled pre-miR-control, pre-miR-135b, anti-miR-control or anti-miR-135b. **(B and C)** Western blot analysis of the TGFBR2 protein levels in HT-29 and SW-480 cells treated with pre-miR-control, pre-miR-135b, anti-miR-control or anti-miR-135b. B: representative image; C: quantitative analysis. **(D)** Quantitative RT-PCR analysis of the TGFBR2 mRNA levels in HT-29 and SW-480 cells treated with pre-miR-control, pre-miR-135b, anti-miR-control or anti-miR-135b. **(E)** Direct binding of the TGFBR2 3’-UTR to miR-135b. HT-29 and SW-480 cells were co-transfected with a firefly luciferase reporter containing either wild-type (WT) or mutant (Mut) miR-135b binding sites in the TGFBR2 3’-UTR and either pre-miR-control or pre-miR-135b. Twenty-four hours after transfection, the cells were assessed using a luciferase assay kit. The results are displayed as the ratio of firefly luciferase activity in the miR-135b-transfected cells to that in the control cells. * P < 0.05; ** P < 0.01; *** P < 0.001.

The correlation between miR-135b and TGFBR2 expression was further examined by evaluating TGFBR2 expression in the human CRC cell lines HT-29 and SW-480 after the overexpression and knockdown of miR-135b. In these experiments, miR-135b overexpression was achieved by transfecting HT-29 and SW-480 cells with pre-miR-135b, which is a synthetic RNA oligonucleotide that mimics the miR-135b precursor; whereas miR-135b knockdown was achieved by transfecting cells with anti-miR-135b (chemically modified antisense oligonucleotides designed to specifically target mature miR-135b). The efficient overexpression or knockdown of miR-135b is demonstrated in Fig 3A. Clearly, cellular miR-135b levels were significantly increased when HT29 and SW480 cells were transfected with pre-miR-135b and dropped dramatically when HT29 and SW480 cells were treated with anti-miR-135b. The expression of TGFBR2 protein was significantly inhibited by the introduction of pre-miR-135b in HT29 (Fig 3B) and SW480 (Fig 3C) cells, while anti-miR-135b significantly increased the TGFBR2 protein level in HT29 (Fig 3B) and SW480 cells (Fig 3C). To determine the level at which miR-135b regulates TGFBR2 expression, we repeated the above experiments and examined the expression of TGFBR2 mRNA after transfection. The overexpression or knockdown of miR-135b did not affect the mRNA stability of TGFBR2 (Fig 3D). These results demonstrated that miR-135b specifically regulates TGFBR2 expression at the post-transcriptional level, which is the most common mechanism of animal miRNA action.

To determine whether the negative regulatory effects of miR-135b on TGFBR2 expression were mediated by the binding of miR-135b to the predicted target sites in the 3’-UTR of the TGFBR2 mRNA, the full length 3’-UTR of TGFBR2 containing the predicted miR-135b binding site was inserted downstream of the firefly luciferase gene in a reporter plasmid. The resulting plasmid was co-transfected into HT-29 and SW-480 cells with a transfection control plasmid (β-gal) and either pre-miR-135b or a scrambled negative control RNA. As expected, the luciferase activity was markedly reduced in the cells transfected with pre-miR-135b. Furthermore, we introduced point mutations into the corresponding complementary sites in the 3’-UTR of TGFBR2 to disrupt the predicted miR-135b binding sites. This mutated luciferase reporter was unaffected by the overexpression of miR-135b (Fig 3E). This finding suggested that the putative miRNA binding sites of TGFBR2 strongly contribute to this miRNA-mRNA interaction, which mediates the post-transcriptional repression of TGFBR2 expression. In conclusion, our results demonstrated that miR-135b directly binds to the 3’-UTR of the TGFBR2 mRNA transcript to suppress TGFBR2 expression in CRC cells.

### miR-135b promotes the proliferation and inhibits the apoptosis of CRC cells by targeting TGFBR2

**Fig 4:**
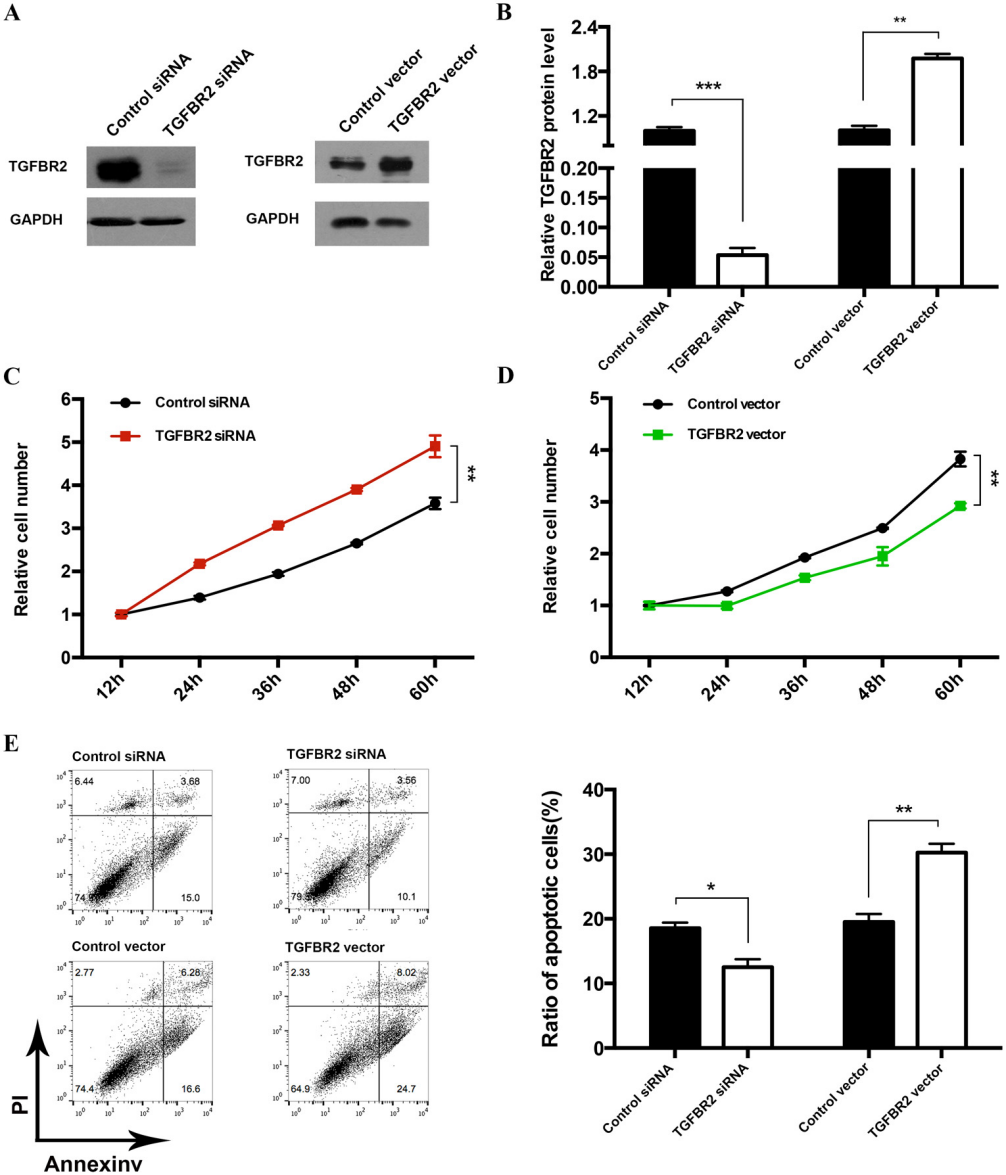
Effects of TGFBR2 expression on the proliferation and apoptosis of HT-29 cells. **(A and B)** Western blot analysis of the TGFBR2 protein levels in HT-29 cells treated with scrambled control siRNA, TGFBR2 siRNA, control vector or TGFBR2 vector. A: representative image; B: quantitative analysis. **(C)** CCK8 cell viability assays were performed 12, 24, 36, 48 and 60 h after the transfection of HT-29 cells with either scrambled control siRNA or TGFBR2 siRNA. **(D)** CCK8 cell viability assays were performed 12, 24, 36, 48 and 60 h after the transfection of HT-29 cells with either control vector or TGFBR2 vector. **(E)** Apoptosis assays were performed after the transfection of HT-29 cells with scrambled control siRNA, TGFBR2 siRNA, control vector or TGFBR2 vector. Left panel: representative image; right panel: quantitative analysis. * P < 0.05; ** P < 0.01.

We next analyzed the biological consequences of the miR-135b-driven repression of TGFBR2 expression in CRC cells. We first investigated whether the overexpression or silencing of TGFBR2 affects HT-29 cell proliferation and apoptosis. To knock down TGFBR2 expression, the siRNA sequence targeting different sites of human TGFBR2 cDNA was designed and transfected into HT-29 cells. To overexpress TGFBR2, a plasmid expressing the TGFBR2 ORF was transfected into HT-29 cells. The efficient overexpression of TGFBR2 is demonstrated in Fig 4A and 4B. Consistent with previous studies showing that TGFBR2 inhibits cell proliferation [3], HT-29 cells in which TGFBR2 expression was knocked down using siRNA proliferated at a significantly higher rate, whereas the cells transfected with the TGFBR2 overexpression plasmid exhibited significantly reduced proliferation (Fig 4C and 4D). Similarly, HT-29 cells in which TGFBR2 expression was knocked down using siRNA exhibited significantly increased apoptosis, whereas those transfected with the TGFBR2 overexpression plasmid exhibited reduced apoptosis (Fig 4E).

**Fig 5:**
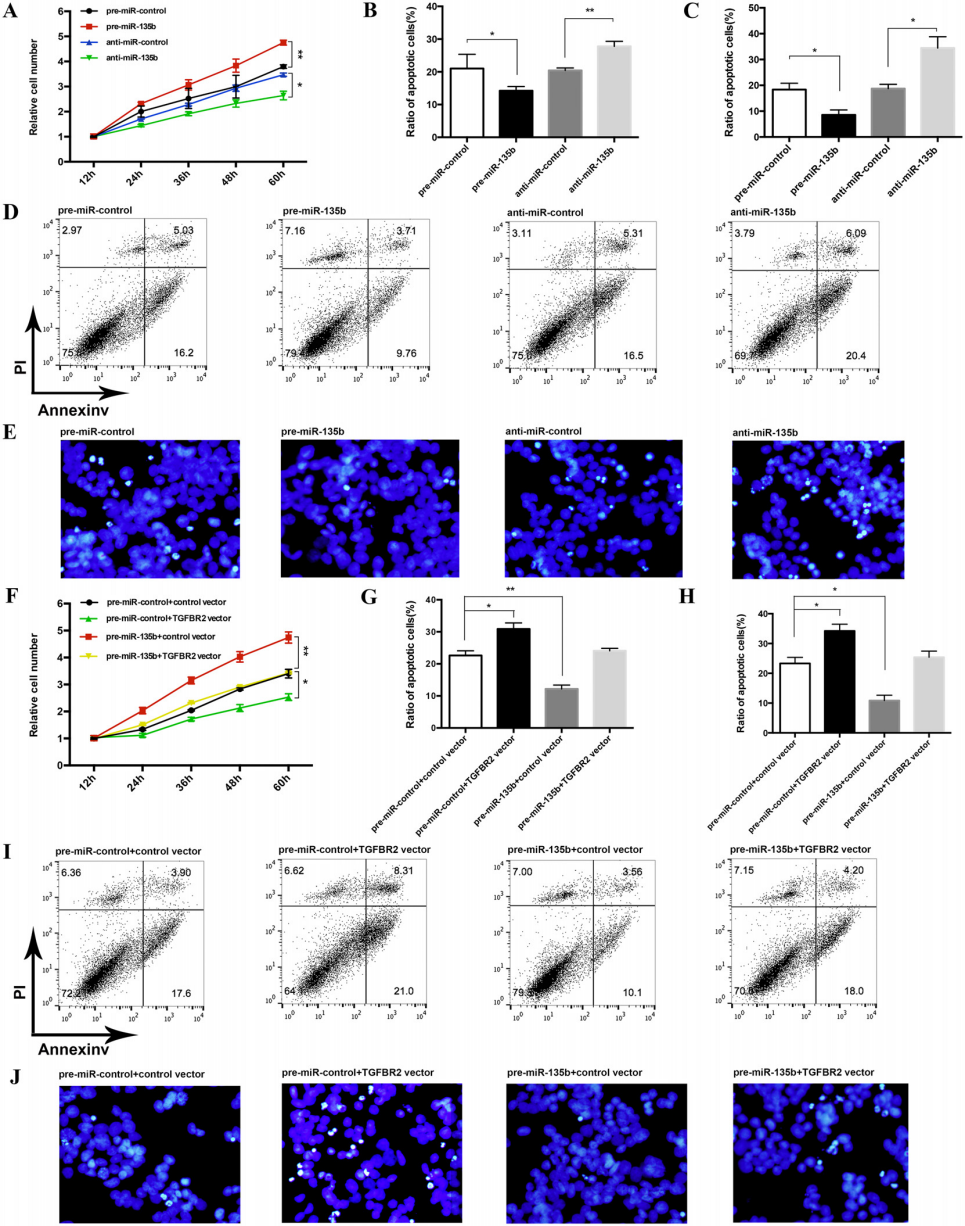
The effect of miR-135b on the proliferation and apoptosis of CRC cells. **(A)** CCK-8 cell viability assays were performed 12, 24, 36, 48 and 60 h after the transfection of HT-29 cells with pre-miR-control, pre-miR-135b, anti-miR-control or anti-miR-135b. **(B-E)** Apoptosis assays were performed 24 h after the transfection of HT-29 cells with pre-miR-control, pre-miR-135b, anti-miR-control or anti-miR-135b. B: representative percentage of apoptotic HT-29 cells using flow cytometric analysis; C: representative percentage of apoptotic HT-29 cells using DAPI staining; D: representative image using flow cytometric analysis; C: representative image of apoptotic HT-29 cells using DAPI staining. * P < 0.05; ** P < 0.01. **(F)** CCK-8 cell viability assays were performed 12, 24, 36, 48 and 60 h after the transfection of HT-29 cells with the scrambled control miRNA and the control vector, the scrambled control miRNA and the TGFBR2 vector, pre-miR-135b and the control vector, or pre-miR-135b and the TGFBR2 vector. **(G-J)** Apoptosis assays were performed 24 h after the transfection of HT-29 cells with the scrambled control miRNA and the control vector, the scrambled control miRNA and the TGFBR2 vector, pre-miR-135b and the control vector, or pre-miR-135b and the TGFBR2 vector. G: representative percentage of apoptotic HT-29 cells using flow cytometric analysis; H: representative percentage of apoptotic HT-29 cells using DAPI staining; I: representative image using flow cytometric analysis; J: representative image of apoptotic HT-29 cells using DAPI staining. * P < 0.05; ** P < 0.01.

We next analyzed the biological consequences of the miR-135b-mediated suppression of TGFBR2 expression in CRC cells. In support of the notion that miR-135b functions as a key proto-oncogenic miRNA [32], HT-29 cells transfected with pre-miR-135b exhibited increased proliferation; in contrast, knockdown of miR-135b had the opposite effect on cell proliferation (Fig 5A). Moreover, compared to the cells transfected with pre-miR-135b, the cells co-transfected with pre-miR-135b and the TGFBR2 overexpression plasmid exhibited a significantly reduced proliferation rate (Fig 5F), suggesting that miR-135b—resistant TGFBR2 expression rescued the suppression of TGFBR2 expression by miR-135b and attenuated the pro-proliferative effect of miR-135b.

We investigated the effects of miR-135b on CRC cell apoptosis via flow cytometric analysis and DAPI staining. The results showed that the percentage of apoptotic cells was significantly lower among the HT-29 cells transfected with pre-miR-135b but was higher among the HT-29 cells transfected with anti-miR-135b (Fig 5B, 5C, 5D and 5E). Furthermore, when HT-29 cells were simultaneously transfected with pre-miR-135b and the TGFBR2 overexpression plasmid, the anti-apoptotic effect of miR-135b was dramatically attenuated (Fig 5G, 5H, 5I and 5J). These results indicated that miR-135b might modulate cell apoptosis by downregulating TGFBR2 in CRC cells.

## Discussion

The TGF-β signaling pathway plays a complex role in the malignant progression of tumors, and its effects, such as growth inhibition, apoptosis, differentiation and other TGF-β-regulated processes, appear to be context-dependent [33]. TGFBR2 expression is required for TGF-β signaling to regulate the specificity of its signaling pathway activation and its biological effects [34]. TGFBR2 acts as a tumor suppressor, and the loss of TGFBR2 function may be an important determinant in the malignant transformation of intestinal neoplasms initiated by APC [35]. The reduction or loss of TGFBR2 expression enables cancer cells to escape the growth inhibitory effect of TGF-β and to gain a proliferative advantage, and the downregulation of TGFBR2 is a frequent event in early-stage CRC. The mechanism of TGFBR2 deregulation varies in different types of cancer [9,36]. The loss of TGFBR2 expression may be attributed to defects in either genetic or epigenetic events. Analysis of the mechanism underlying TGFBR2 loss may provide clinical value for the identification of new therapeutic targets and approaches for certain subsets of cancer. In this study, we found an alternative mechanism that regulates TGFBR2 expression at the post-transcriptional level in CRC.

In this study, we predicted that TGFBR2 is a target of miR-135b, a miRNA that has been reported in many studies to be upregulated and to function as an oncogenic unit in CRC [21,32,37,38]. In 2006, Bandres et al.[21] firstly found that miR-135b were significantly upregulated in CRC tissues and cell lines. Nagel et al.[37] uncovered a miR-135 family-mediated mechanism for colorectal cancer pathogenesis by regulating APC expression and Wnt pathway activity. Furthermore, Wu et al.[38] found that miR-135b may involve CRC progression via downregulating MTSS1 expression and contributing to CRC cell invasion. Recently, Valeri et al.[32] revealed the role of miR-135b in promoting CRC progression as a downstream effector of oncogenic pathways. After measuring the expression levels of miR-135b and TGFBR2 in paired human CRC and noncancerous tissue, we detected an inverse correlation between the miR-135b levels and the TGFBR2 protein levels. Furthermore, by overexpressing or silencing miR-135b in CRC cells, we experimentally validated that miR-135b directly inhibits TGFBR2 translation. Finally, we showed that miR-135b inhibits TGFBR2 expression and consequently promotes the proliferation and inhibits the apoptosis of CRC cells. These results delineate a novel regulatory network by which miR-135b and TGFBR2 regulate cell proliferation and apoptosis. We also provided evidence that the restoration of TGFBR2 expression reverses miR-135b-induced cell proliferation and anti-apoptotic effects, suggesting that the targeting of TGFBR2 is one mechanism by which the miR-135b exerts its proto-oncogenic effect. Therefore, the modulation of TGFBR2 by miR-135b may explain why the upregulation of miR-135b during CRC carcinogenesis promotes cancer progression.

In this study, we found that miR-135b was highly expressed in CRC tissues compared with normal noncancerous tissues. These results suggested that miR-135b may act as an oncogene to participate in the pathogenesis of CRC. Several targets of miR-135b that play vital roles in CRC and other types of cancer have been extensively investigated in previous studies [37,38]. MiR-135b has been frequently reported to be overexpressed in CRC tissues and cell lines [21,39]. A recent study also demonstrated that miR-135b might be a key downstream effector of oncogenic pathways, such as the Wnt and PTEN/PI3K signaling pathways, and might serve as a potential target for CRC treatment [32].

Our data suggest that elevated miR-135b expression is another modulator of the TGF-β pathway that may cooperate with the currently known mechanisms underlying TGFBR2 deregulation, including the accumulation of TGFBR2 mutations or promoter methylation.

## Supporting Information

S1 Table Clinical features of colorectal cancer patients. (DOC)

## References

[1] Jemal A, Bray F, Center MM, Ferlay J, Ward E, et al (2011) Global cancer statistics. CA: A Cancer Journal for Clinicians 61: 69–90. 10.3322/caac.20107 21296855

[2] Muñoz NM, Upton M, Rojas A, Washington MK, Lin L, et al (2006) Transforming Growth Factor β Receptor Type II Inactivation Induces the Malignant Transformation of Intestinal Neoplasms Initiated by Apc Mutation. Cancer Research 66: 9837–9844. 17047044 10.1158/0008-5472.CAN-06-0890

[3] Yu M, Trobridge P, Wang Y, Kanngurn S, Morris SM, et al (2014) Inactivation of TGF-beta signaling and loss of PTEN cooperate to induce colon cancer in vivo. Oncogene 33: 1538–1547. 10.1038/onc.2013.102 23604118 PMC3883899

[4] Biswas S, Chytil A, Washington K, Romero-Gallo J, Gorska AE, et al (2004) Transforming growth factor beta receptor type II inactivation promotes the establishment and progression of colon cancer. Cancer Res 64: 4687–4692. 15256431 10.1158/0008-5472.CAN-03-3255

[5] Moustakas A, Heldin CH (2009) The regulation of TGFbeta signal transduction. Development 136: 3699–3714. 10.1242/dev.030338 19855013

[6] Massague J, Blain SW, Lo RS (2000) TGFbeta signaling in growth control, cancer, and heritable disorders. Cell 103: 295–309. 11057902 10.1016/s0092-8674(00)00121-5

[7] Engle SJ, Hoying JB, Boivin GP, Ormsby I, Gartside PS, et al (1999) Transforming growth factor beta1 suppresses nonmetastatic colon cancer at an early stage of tumorigenesis. Cancer Res 59: 3379–3386. 10416598

[8] Gryfe R, Swallow C, Bapat B, Redston M, Gallinger S, et al (1997) Molecular biology of colorectal cancer. Curr Probl Cancer 21: 233–300. 9438104 10.1016/s0147-0272(97)80003-7

[9] Zhang Q, Rubenstein JN, Jang TL, Pins M, Javonovic B, et al (2005) Insensitivity to transforming growth factor-beta results from promoter methylation of cognate receptors in human prostate cancer cells (LNCaP). Mol Endocrinol 19: 2390–2399. 15905358 10.1210/me.2005-0096

[10] Dong Z, Guo W, Guo Y, Kuang G, Yang Z (2012) Concordant promoter methylation of transforming growth factor-beta receptor types I and II occurs early in esophageal squamous cell carcinoma. Am J Med Sci 343: 375–381. 10.1097/MAJ.0b013e3182253430 22314103

[11] Guo W, Dong Z, Guo Y, Kuang G, Yang Z, et al (2012) Concordant repression and aberrant methylation of transforming growth factor-beta signaling pathway genes occurs early in gastric cardia adenocarcinoma. Mol Biol Rep 39: 9453–9462. 10.1007/s11033-012-1810-x 22722999

[12] Markowitz S, Wang J, Myeroff L, Parsons R, Sun L, et al (1995) Inactivation of the type II TGF-beta receptor in colon cancer cells with microsatellite instability. Science 268: 1336–1338. 7761852 10.1126/science.7761852

[13] Parsons R, Myeroff LL, Liu B, Willson JK, Markowitz SD, et al (1995) Microsatellite instability and mutations of the transforming growth factor beta type II receptor gene in colorectal cancer. Cancer Res 55: 5548–5550. 7585632

[14] Ogino S, Kawasaki T, Ogawa A, Kirkner GJ, Loda M, et al (2007) TGFBR2 mutation is correlated with CpG island methylator phenotype in microsatellite instability-high colorectal cancer. Hum Pathol 38: 614–620. 17270239 10.1016/j.humpath.2006.10.005

[15] Liu B, Farrington SM, Petersen GM, Hamilton SR, Parsons R, et al (1995) Genetic instability occurs in the majority of young patients with colorectal cancer. Nat Med 1: 348–352. 7585065 10.1038/nm0495-348

[16] Bartel DP (2004) MicroRNAs: genomics, biogenesis, mechanism, and function. Cell 116: 281–297. 14744438 10.1016/s0092-8674(04)00045-5

[17] Ambros V (2004) The functions of animal microRNAs. Nature 431: 350–355. 15372042 10.1038/nature02871

[18] He L, Hannon GJ (2004) MicroRNAs: small RNAs with a big role in gene regulation. Nat Rev Genet 5: 522–531. 15211354 10.1038/nrg1379

[19] Calin GA, Sevignani C, Dumitru CD, Hyslop T, Noch E, et al (2004) Human microRNA genes are frequently located at fragile sites and genomic regions involved in cancers. Proceedings of the National Academy of Sciences of the United States of America 101: 2999–3004. 14973191 10.1073/pnas.0307323101 PMC365734

[20] Volinia S, Calin GA, Liu CG, Ambs S, Cimmino A, et al (2006) A microRNA expression signature of human solid tumors defines cancer gene targets. Proc Natl Acad Sci U S A 103: 2257–2261. 16461460 10.1073/pnas.0510565103 PMC1413718

[21] Bandres E, Cubedo E, Agirre X, Malumbres R, Zarate R, et al (2006) Identification by Real-time PCR of 13 mature microRNAs differentially expressed in colorectal cancer and non-tumoral tissues. Mol Cancer 5: 29 16854228 10.1186/1476-4598-5-29 PMC1550420

[22] Esquela-Kerscher A, Slack FJ (2006) Oncomirs—microRNAs with a role in cancer. Nat Rev Cancer 6: 259–269. 16557279 10.1038/nrc1840

[23] Calin GA, Croce CM (2006) MicroRNA signatures in human cancers. Nat Rev Cancer 6: 857–866. 17060945 10.1038/nrc1997

[24] Iorio MV, Ferracin M, Liu CG, Veronese A, Spizzo R, et al (2005) MicroRNA gene expression deregulation in human breast cancer. Cancer Res 65: 7065–7070. 16103053 10.1158/0008-5472.CAN-05-1783

[25] Michael MZ, SM OC, van Holst Pellekaan NG, Young GP, James RJ (2003) Reduced accumulation of specific microRNAs in colorectal neoplasia. Mol Cancer Res 1: 882–891. 14573789

[26] Chen X, Guo X, Zhang H, Xiang Y, Chen J, et al (2009) Role of miR-143 targeting KRAS in colorectal tumorigenesis. Oncogene 28: 1385–1392. 10.1038/onc.2008.474 19137007

[27] Kent OA, Fox-Talbot K, Halushka MK (2013) RREB1 repressed miR-143/145 modulates KRAS signaling through downregulation of multiple targets. Oncogene 32: 2576–2585. 10.1038/onc.2012.266 22751122 PMC8177721

[28] Pagliuca A, Valvo C, Fabrizi E, di Martino S, Biffoni M, et al (2013) Analysis of the combined action of miR-143 and miR-145 on oncogenic pathways in colorectal cancer cells reveals a coordinate program of gene repression. Oncogene 32: 4806–4813. 10.1038/onc.2012.495 23128394

[29] Lewis BP, Shih IH, Jones-Rhoades MW, Bartel DP, Burge CB (2003) Prediction of mammalian microRNA targets. Cell 115: 787–798. 14697198 10.1016/s0092-8674(03)01018-3

[30] John B, Enright AJ, Aravin A, Tuschl T, Sander C, et al (2004) Human MicroRNA targets. PLoS Biol 2: e363 15502875 10.1371/journal.pbio.0020363 PMC521178

[31] Krek A, Grun D, Poy MN, Wolf R, Rosenberg L, et al (2005) Combinatorial microRNA target predictions. Nat Genet 37: 495–500. 15806104 10.1038/ng1536

[32] Valeri N, Braconi C, Gasparini P, Murgia C, Lampis A, et al (2014) MicroRNA-135b Promotes Cancer Progression by Acting as a Downstream Effector of Oncogenic Pathways in Colon Cancer. Cancer Cell 25: 469–483. 10.1016/j.ccr.2014.03.006 24735923 PMC3995091

[33] de Caestecker MP, Piek E, Roberts AB (2000) Role of transforming growth factor-beta signaling in cancer. J Natl Cancer Inst 92: 1388–1402. 10974075 10.1093/jnci/92.17.1388

[34] Rojas A, Padidam M, Cress D, Grady WM (2009) TGF-beta receptor levels regulate the specificity of signaling pathway activation and biological effects of TGF-beta. Biochim Biophys Acta 1793: 1165–1173. 10.1016/j.bbamcr.2009.02.001 19339207 PMC2700179

[35] MacKay SL, Auffenberg T, Tannahill CL, Ksontini R, Josephs MD, et al (1998) Transfection of the type II TGF-beta receptor into colon cancer cells increases receptor expression, inhibits cell growth, and reduces the malignant phenotype. Ann Surg 227: 781–789. 9637541 10.1097/00000658-199806000-00001 PMC1191376

[36] Hougaard S, Nørgaard P, Abrahamsen N, Moses HL, Spang-Thomsen M, et al (1999) Inactivation of the transforming growth factor β type II receptor in human small cell lung cancer cell lines. British Journal of Cancer 79: 1005–1011. 10098728 10.1038/sj.bjc.6690161 PMC2362261

[37] Nagel R, le Sage C, Diosdado B, van der Waal M, Oude Vrielink JA, et al (2008) Regulation of the adenomatous polyposis coli gene by the miR-135 family in colorectal cancer. Cancer Res 68: 5795–5802. 10.1158/0008-5472.CAN-08-0951 18632633

[38] Wu W, Wang Z, Yang P, Yang J, Liang J, et al (2014) MicroRNA-135b regulates metastasis suppressor 1 expression and promotes migration and invasion in colorectal cancer. Mol Cell Biochem 388: 249–259. 10.1007/s11010-013-1916-z 24343340

[39] Gaedcke J, Grade M, Camps J, Sokilde R, Kaczkowski B, et al (2012) The rectal cancer microRNAome—microRNA expression in rectal cancer and matched normal mucosa. Clin Cancer Res 18: 4919–4930. 22850566 10.1158/1078-0432.CCR-12-0016 PMC6298229

