# Correction: miR-135b Promotes Cancer Progression by Targeting Transforming Growth Factor Beta Receptor II (TGFBR2) in Colorectal Cancer

**DOI:** 10.1371/journal.pone.0145589

**Published:** 2015-12-17

**Authors:** Jialu Li, Hongwei Liang, Ming Bai, Tao Ning, Cheng Wang, Qian Fan, Yanbo Wang, Zheng Fu, Nan Wang, Rui Liu, Ke Zen, Chen-Yu Zhang, Xi Chen, Yi Ba

The affiliation for the fourteenth author is incorrect. Yi Ba is not affiliated with #2 but with #1Tianjin Medical University Cancer Institute and Hospital, Key Laboratory of Cancer Prevention and Therapy, Tiyuanbei, Tianjin, 300060, China.
