# Supplementary material for: miR-135b Promotes Cancer Progression by Targeting Transforming Growth Factor Beta Receptor II (TGFBR2) in Colorectal Cancer
Source: PLoS One. 2015 Jun 10;10(6):e0130194. doi: 10.1371/journal.pone.0130194 (PMC4462589; doi:10.1371/journal.pone.0130194)
Supplement: S1 Table — (DOC) [file pone.0130194.s001.doc]

**Supporting Information**

**miR-135b promotes cancer progression by targeting transforming growth factor beta receptor II (TGFBR2) in colorectal cancer**

**S1 Table.** Clinical features of colorectal cancer patients.

|  | Age | Gender | Pathological  Stage | Tumor  Histotype |
| --- | --- | --- | --- | --- |
| 1 | 62 | M | III(T3,N1,M0) | Adenocarcinoma |
| 2 | 55 | F | III(T3,N1,M0) | Adenocarcinoma |
| 3 | 68 | M | II(T3,N0,M0) | Adenocarcinoma |
| 4 | 63 | M | II(T4,N0,M0) | Adenocarcinoma |
| 5 | 51 | F | III(T4,N2,M0) | Adenocarcinoma |
